# Correction of the significance level when attempting multiple transformations of an explanatory variable in generalized linear models

**DOI:** 10.1186/1471-2288-13-75

**Published:** 2013-06-08

**Authors:** Benoit Liquet, Jérémie Riou

**Affiliations:** 1 University Bordeaux, ISPED, Centre INSERM U-897-Epidemiologie-Biostatistique, Bordeaux, F-33000, France; 2 INSERM, ISPED, Centre INSERM U-897-Epidemiologie-Biostatistique, Bordeaux, F-33000, France; 3 MRC Biostatistics Unit, Institute of Public Health, Cambridge, CB2 0SR, UK; 4 Danone Research, Avenue de la Vauve, Route départementale 128, Palaiseau Cedex 91767, France

**Keywords:** Bonferroni procedure, Generalized linear model, Multiple coding, Parametric bootstrap, Permutation, *p*_*value*_, Resampling procedure

## Abstract

**Background:**

In statistical modeling, finding the most favorable coding for an exploratory quantitative variable involves many tests. This process involves multiple testing problems and requires the correction of the significance level.

**Methods:**

For each coding, a test on the nullity of the coefficient associated with the new coded variable is computed. The selected coding corresponds to that associated with the largest statistical test (or equivalently the smallest *p*_*value*_). In the context of the Generalized Linear Model, Liquet and Commenges (Stat Probability Lett,71:33–38,2005) proposed an asymptotic correction of the significance level. This procedure, based on the score test, has been developed for dichotomous and Box-Cox transformations. In this paper, we suggest the use of resampling methods to estimate the significance level for categorical transformations with more than two levels and, by definition those that involve more than one parameter in the model. The categorical transformation is a more flexible way to explore the unknown shape of the effect between an explanatory and a dependent variable.

**Results:**

The simulations we ran in this study showed good performances of the proposed methods. These methods were illustrated using the data from a study of the relationship between cholesterol and dementia.

**Conclusion:**

The algorithms were implemented using R, and the associated CPMCGLM R package is available on the CRAN.

## Background

In applied studies, the relationship between an explanatory and a dependent variable is routinely measured using a statistical model. For instance, in epidemiology it is quite common that a study focuses on one particular risk factor. The scientific problem is to analyze whether this risk factor has an influence on the risk of occurrence of a disease, a biological trait, or another outcome. To answer to this question, a regression model is often used in which the risk factor will be represented by a continuous *X*, allowing adjustment on *p*−1 known risk factors of the studied trait. However, the form of the effect (or the dose-effect relationship) is not known in advance, and as such, the continuous variable *X* is often transformed, typically into categorical variables, by grouping values into two or more categories. An example of this is seen in an *The American Journal of Epidemiology* (October 2009, volume 170, number 8), where four of six papers with continuous exposure used categorization, and only two kept the variable as continuous [1].

Binary coding is often used in epidemiology, either to make interpretation easier, or because a threshold effect is suspected. In a regression model with multiple explanatory variables, the interpretation of the regression coefficient for a binary variable may be easier to understand than a change in one unit of the continuous variable. Dichotomous transformations of a variable *X* are defined as:

$$X(k)=\left\{\begin{array}{cc} 1 & \;\text{if}\;X\geq c_{k}\\ 0 & \;\text{if}\;X<c_{k} \end{array}\right)$$

Other transformations are also used, in particular Box-Cox transformations which have been defined as:

$$X(k)=\left\{\begin{array}{ll} \lambda_{k}^{-1}(X^{\lambda_{k}}-1) & \;\text{if}\;\lambda_{k}>0\\ \log X & \;\text{if}\;\lambda_{k}=0\text{,} \end{array}\right)$$

but the choice of the transformation is often subjective. The arbitrariness of the choice of cutpoints may lead to the idea of trying more than one set of values. Hence to analyze data, the statistician may have to use several transformations, and for each the statistician applies a test for “ *β*=0” (where *β* is the coefficient representing the effect of the risk factor of interest). The most favorable transformation is then chosen. The cutpoint giving the minimum *p*_*value*_ is often termed “optimal” [2,3]. When testing several codings of a variable, there is a problem with the multiplicity of tests performed, leading to an incorrect *p*_*value*_ and possible overestimation of effects [4]. Generally, researchers fail to consider this problem and do not correct the significance level in relation to the number of tests performed [3], which can lead to an increase in the Type-I error [5]. The *p*_*value*_ should thus be corrected to take into account the multiplicity of tests.

In many cases, it is now widely recognized that categorization of a continuous variable could introduce major problems to an analysis and interpretation of the associated model [1,3]. It is important to note that the aim of this paper is not to defend this practice, but to improve a practice commonly used by epidemiologists in terms of multiple testing. Furthermore, despite known loss of power following dichotomization in the univariate case, Westfall [6] shown that dichotomizing continuous data can greatly improve the power when multiple comparisons are performed.

Many methods of correction exist, the most simple and well known being the Bonferroni rule. Several authors have improved this method to make it more powerful, however most do not take into account the correlation between the tests [7-11]. If the tests are independent, or moderately dependent, then they provide an upper bound which may be satisfactory. Efron [12] proposed a correction that account for the correlation between two consecutive tests if there is a natural order between the tests, with high correlation between adjacent tests. Liquet and Commenges [13,14] and Hashemi and Commenges [15] proposed a more exact correction, accounting for the whole correlation matrix, for score tests obtained in logistic regression, generalized linear model and proportional hazards models.

Here, we proprose extending these studies to a categorical transformation (with *m*>2 categories) of the continuous variable by involving more than one parameter in the model; *m*−1 dummy variables are introduced in the model. The categorical transformation is a more flexible way to explore the unknown shape of the effect. In this context, we propose a method and an R program based on resampling approaches to determine the significance level for a series of several transformations (including dichotomous, Box-Cox and categorical transformations) of an explanatory variable in a Generalized Linear Model. The problem of correcting the estimation of the effect will not be examined here.

First, we revisit the example proposed by Liquet and Commenges [14] on the relationship between cholesterol and dementia [16] to provide a framework for our discussion. In section ‘Methods: Statistical context’, we present the statistical contexts relating to multiple testing; the model, the maximum test and the minimum *p*_*value*_ procedure and finally the score tests are exposed. Section ‘Methods: Significance level correction’ presents the different methods of correction of the Type-I error. A simulation study for the different strategies of coding, and application of the model to the initial example are presented in the section ‘Results’. Concluding remarks are given in the two last sections.

## Example: revisiting the PAQUID cohort example

We revisited the example presented in the article of Liquet and Commenges [13] for a coding of a binary variable in a logistic regression. This example is based on the work of Bonarek et al. [16], who studied the relationship between serum cholesterol levels and dementia. The data came from a nested case-control study of 334 elderly French subjects aged 73 and over who participated in the PAQUID cohort (37 subjects with dementia and 297 controls). The variables age, sex, level of education and wine consumption were considered as adjustment variables. The analysis focused on the influence of HDL-cholesterol(high-density lipoprotein) on the risk of dementia. Bonarek et al. [16] first considered HDL-cholesterol as a continuous variable; then, to ease clinical interpretation, they chose to transform the HDL-cholesterol into a categorical variable with four classes. Finally, as there was no significant difference between the first three quartiles, HDL-cholesterol was split into two categories with a cutpoint at the last quartile. The best *p*_*value*_, 0.007, was obtained in the latter analysis and was selected for interpretation. However, this *p*_*value*_ did not take into account the numerous transformations performed to determine the best representation of the variable of interest. Legitimate questions arising from this include the following: What is the real association between dementia and HDL-cholesterol, with a correction of the Type-I error? Is it really significant? Liquet and Commenges [14] proposed correcting the *p*_*value*_ associated with multiple transformation including dichotomous and Box-Cox transformation, however, their method cannot be used with categorical transformation.

## Methods

### Statistical context

#### Model

Let us consider a Generalized Linear Model with *p* explanatory variables [17], where *Y*_*i*_ (1≤*i*≤*n*) are independently distributed with probability density function in the exponential family defined as follows:

(1) $$f_{Y_{i}}(y_{i},\theta_{i},\phi )=\text{exp}\left\{\frac{y_{i}\theta_{i}-b(\theta_{i})}{a(\phi )}+c(y_{i},\phi )\right\};$$

with $E[Y_{i}]=\mu_{i}=b^{\prime }(\theta_{i}),V\text{ar}[Y_{i}]=b^{\prime \prime }(\theta_{i})a(\phi )$ and where *a*(·), *b*(·), and *c*(·) are known and differentiable functions. *b*(·) is three times differentiable, and its first derivative *b*^′^(·) is invertible. Parameters (*θ*_*i*_,*ϕ*) belong to $\Omega \subset {\mathbb{R}}^{2}$, where *θ*_*i*_ is the canonical parameter and *ϕ* is the dispersion parameter.

In this context, we wished to test the association between the outcome *Y*_*i*_ and explanatory variable of interest *X*_*i*_, adjusted on a vector of explanatory variables *Z*_*i*_. The form of the effect of *X*_*i*_ is unknown, so we may consider *K* transformations of this variable *X*_*i*_**(k)**=*g*_*k*_(*X*_*i*_) with *k*=1,…,*K*.For example, if we transform the continuous variable in *m*_*k*_ classes, *m*_*k*_−1 dummy variables are defined from the function *g*_*k*_(·): $X_{i}(k)=g_{k}(X_{i})=(X_{i}^{1}(k),\ldots ,X_{i}^{m_{k}-1}(k))$. Different numbers of level *m*_*k*_ of the categorical transformation are possible.The model for a transformation *k* can then be obtained by modeling the canonical parameter *θ*_*i*_ as:

(2) $$\theta_{i}(k)=\gamma Z_{i}+\beta_{k}X_{i}(k),\;\;i=1,\ldots ,n;$$

where $Z_{i}=(1,Z_{i}^{1},\ldots ,Z_{i}^{p-1})$ and ***γ***=(*γ*_0_,…,*γ*_*p*−1_)^*T*^ is a *p*−1 vector of coefficients, and ***β***_***k***_ is the *m*_*k*_−1 vector of coefficients associated with a categorical transformation *k* of the variable *X*_*i*_. For dichotomous or Box-Cox transformations ***β***_***k***_ reduce to a scalar ($\beta_{k}\in \mathbb{R}$).The hypothesis of the test for the transformation *k* is defined as follows:

$${ℋ}_{0}(k):\beta_{k}=0_{m_{k}-1}\;\;\text{versus}\;\;{ℋ}_{1}(k):\beta_{k}\neq 0_{m_{k}-1},$$

where $0_{m_{k}-1}$ is a null vector of dimension *m*_*k*_−1. Under the null hypothesis ${ℋ}_{0}(k)$ we have *θ*_*i*_(*k*)=***γ****Z*_*i*_, which do not depend on *k*. Thus all the null hypotheses are the same, and denote it by ${ℋ}_{0}$.

#### Maximum test and minimum P-value procedures

For each coding, *k*, of the variable *X*_*i*_, a test statistic *T*_*k*_ is performed on the nullity of the vector ***β***_***k***_. We then have a vector of test statistics **T**=(*T*_1_,…,*T*_*K*_) for the same null hypothesis (no effect of the risk factor of interest). In the context of dichotomous and Box-Cox transformations, each test statisitic, *T*_*k*_, has asymptotically, a standard normal distribution. Thus rejecting the null hypothesis if one of the absolute values of the test *T*_*k*_ is larger than a critical value *c*_*α*_, is equivalent to rejecting the null hypothesis if *T*_*max*_>*c*_*α*_ where *T*_*max*_=*max*(|*T*_1_|,…,|*T*_*K*_|). To cope with the multiplicity problem, Liquet and Commenges [13,14] proposed that the probablity of Type-I error for the statistic *T*_*max*_ under the null hypothesis be computed as:

(3) $$\begin{array}{lll} p_{\text{value}} & =P(T_{\text{max}}\geq t_{\text{max}})=1-P(|T_{1}|\; & \;\\ & <t_{\text{max}},\ldots ,|T_{\text{max}}|<t_{\text{max}},)\; \end{array}$$

where *T*_*max*_ is the realization of *T*_*max*_.An equivalent approach is to use a procedure based on the individual *p*_*value*_ of each test *T*_*k*_ noted *P*_*k*_=*P*(|*T*_*k*_|>|*t*_*k*_|) (where *T*_*k*_ is the realization of *T*_*k*_). The minimum of the *K* realized *p*_*value*_ corresponds to the test *k* which obtains the highest realization (in absolute values; *k*/ *t*_*max*_=|*t*_*k*_|). Then, we have:

(4) $$\begin{array}{ll} p_{\text{value}}=P(P_{\text{min}}\leq p_{\text{min}}) & \; \end{array}$$

where *P*_*min*_=*min*(*P*_1_,…,*P*_*K*_) and *p*_*min*_ is the realization of *p*_*min*_. The interest of using a procedure based on the *p*_*value*_ is the possibility of combining statistical tests which do not follow the same distribution. In the current context, we will combine dichotomous, Box-Cox and categorical transformations with more than two levels.

#### Score test

We briefly present the score test used for all of the K transformations where the same null hypothesis is tested (*i.e.*${ℋ}_{0}$: “$\beta_{k}=0_{m_{k}-1}$” given by $\theta_{i}(k)=\gamma Z_{i}$ (with different alternatives)). We present the main results obtained by Liquet and Commenges [14] for the Generalized Linear Model in the context of dichotomous and Box-Cox transformations, and then consider the score test for categorical transformations.

##### Dichotomous and Box-cox Transformations

In the context of dichotomous and Box-Cox transformations, the score test used for testing the effect of the transformed variable (***β***_***k***_=0 with $\beta_{k}\in \mathbb{R}$) follows asymptotically a standard normal distribution:

$$T_{k}=\frac{X{\left(k\right)}^{T}\hat{R}}{\sqrt{X{\left(k\right)}^{T}(I-H)\text{VX}(k)}}$$

where $\hat{R}$ is the vector of residuals $\hat{R_{i}}=Y_{i}-\hat{\mu_{i}}$ computed under the null hypothesis, *V* is a diagonal matrix such that $v_{\text{ii}}=\hat{\text{Var}}(Y_{i}),H=\text{VZ}(Z^{T}\text{VZ})Z^{T}$, and *Z* the *n*×*p* matrix with rows *Z*_*i*_, *i*=1,…,*n*.

The correlation between the different tests has been defined by Liquet and Commenges [14]. Asymptotically, the joint distribution of *T*_1_,…,*T*_*K*_ is a multivariate normal distribution with zero mean and a certain covariance matrix. Thus Liquet and Commenges [14] propose that the *p*_*value*_ (associated with the test *T*_*max*_) defined in (3) using numerical integration [18] be calculated. They called their method the “exact method”.

##### Categorical transformations

In the context of a categorical transformation in *m*_*k*_ classes, the score test testing ${ℋ}_{0}$: “$\beta_{k}=0_{m_{k}-1}$” (with $\beta_{k}\in {\mathbb{R}}^{m_{k}-1}$) follows asymptotically a *χ*^2^ distribution with *m*_*k*_−1 degrees of freedom and is defined as:

$$T_{k}=U_{k}^{T}I_{k}^{-1}U_{k};$$

where *U*_*k*_ and *I*_*k*_ are respectively the score function and the Fisher information matrix under the null hypothesis [19]. To compute the *p*_*value*_ defined in (4), it is necessary to know the joint distribution of **T**=(*T*_1_,…,*T*_*K*_). Some studies have defined the distribution of the multivariate *χ*^2^[20,21]. However, even though the correlation between the different tests could be easily estimated, it has not been possible, as far as we know, to obtain the joint distribution of **T**=(*T*_1_,…,*T*_*K*_). To overcome this problem, we propose approximating the *p*_*value*_ (defined in (4) by the minimum *p*_*value*_ procedure) using a resampling method (defined in the next section) which also accounts for the correlation between the test statistics.

### Significance level correction

#### Bonferroni method

One of the most common corrections in multiple testing is the Bonferroni method. It has been described by several authors in various applications [7,11,22]. It allows an upper bound of the significance level of the minimum *p*_*value*_ procedure to be computed as:

$$p_{\text{value}}=P(P_{\text{min}}\leq p_{\text{min}})\leq K\times p_{\text{min}}$$

where *K* is the number of tests. This method is very simple and does not require any assumption about the correlation between the different tests. It can therefore be applied directly to the different possible codings of an explanatory variable. However, this only provides an upper bound of the *p*_*value*_, which may be very conservative if the correlation between tests are high and the number of transformation are large.

#### Resampling based methods

We propose the use of resampling based methods [23,24] with the aim of building a reference distribution for the test statistics. These procedures have the advantage of taking into account the dependence of the test statistics for evaluating the correct significance level of the minimum *p*_*value*_ procedure (or the maximum test procedure). The principle of resampling procedures is to define new samples from the probability measure defined under ${ℋ}_{0}$: “$\beta_{k}=0_{m_{k}-1}$”.

##### Permutation test procedure

Permutation methods can be used to construct tests which control the Type-I error rate [25]. In our context, the algorithm of the permutation procedure is defined as follows: 

1. Apply the minimum *p*_*value*_ procedure to the original data for the *K* transformations considered. We note *p*_*min*_ the realization of the minimum of the *p*_*value*_;

2. As under ${ℋ}_{0}$, the *X*_*i*_ variable has no effect on the response variable, a new dataset is generated by permuting the *X*_*i*_ variable in the initial dataset;

3. Generate B new datasets $s_{b}^{*},b=1,\ldots ,B$ by repeating *B* times the step 2;

4. For each new dataset, apply the minimum *p*_*value*_ procedure for the transfomation under consideration. We note $p_{\text{min}}^{*b}$ the smallest *p*_*value*_ for each new dataset.

5. The *p*_*value*_ defined in (4) is then approximated by:

$$\hat{p_{\text{value}}}=\frac{1}{B}\overset{B}{\underset{b=1}{\sum }}I_{\left\{p_{\text{min}}^{*b}<p_{\text{min}}\right\}},$$

where *I*_{·}_ is an indicator function.

However, it is important to note that exchangeability need to be satisfied [25-30]. This condition is much more restrictive than it appears at first sight. In fact, Commenges [29] and Commenges and Liquet [25] showed that the permutation test approach for the score test is robust if the model has only one intercept under the null hypothesis, or if *X*_*i*_ are independent of *Z*_*i*_ for all *i* in the context of a linear model and the proportional hazards model. This issue applies in our context. Thus we investigated, the robustness of the permutation method when the exchangeability assumptions is violated.

##### Parametric bootstrap procedure

In 2000, Good [31] explained: “Permutations test hypotheses concerning distributions; bootstraps test hypotheses concerning parameters. As a result, the bootstrap implies less stringent assumptions”. Therefore, an alternative way may be to use resampling method based on bootstrap [32], which give us an asymptotic reference distribution. This procedure could be defined by the following algorithm: 

1. Apply the minimum *p*_*value*_ procedure to the original data for the *K* transformations being considered. We note *p*_*min*_ the realization of the minimum of the *p*_*value*_;

2. Fit the model under the null hypothesis, using the observed data, and obtain $\hat{\gamma }$, the maximum likelihood estimate (MLE) of *γ*;

3. Generate a new outcome $Y_{i}^{*}$ for each subject from the probability measure defined under ${ℋ}_{0}$. For example, for a logistic model (where *a*(*ϕ*)=1, $b(\theta_{i})=\text{log}(1+e^{\theta_{i}})$, and $\mu_{i}=E(Y_{i})=e^{\theta_{i}}/(1+e^{\theta_{i}})$), we generate $Y_{i}^{*}$ according to:

$$P(Y_{i}^{*}=1|Z_{i})=\frac{e^{\hat{\gamma }Z_{i}}}{1+e^{\hat{\gamma }Z_{i}}}.$$

Repeat this for all the subjects to obtain a sample noted $s^{*}=\{Y_{i}^{*},Z_{i},X_{i}\}$

4. Generate B new datasets $s_{b}^{*},b=1,\ldots ,B$ by repeating *B* times the step 3;

5. Apply for each new dataset, the minimum *p*_*value*_ procedure for the transformation considered. We note $p_{\text{min}}^{*b}$ the smallest *p*_*value*_ for each new dataset.

6. Then, the *p*_*value*_ defined in (4) is then approximated by:

$$\hat{p_{\text{value}}}=\frac{1}{B}\overset{B}{\underset{b=1}{\sum }}I_{\left\{p_{\text{min}}^{*b}<p_{\text{min}}\right\}}.$$

## Results

### Simulation study

The aim of this simulation study was to assess the performance of the two resampling methods to correct the significance level. Three different scenarios of transformations were investigated: dichotomous transformations, categorical transformations with three classes, and categorical transformations with different numbers of classes. To shorten the simulation study section we have not presented the results for the Box-Cox transformations. For each simulation case, the control of the Type-I error and the power of the developed methods were evaluated. For all simulations, the data come from a logistic model (where *a*(*ϕ*)=1, $b(\theta_{i})=\text{log}(1+e^{\theta_{i}})$, and $\mu_{i}=E(Y_{i})=e^{\theta_{i}}/(1+e^{\theta_{i}})$) consisting of two explanatory variables: *Z*, an adjustment variable, and *X*, the variable of interest. We considered the following models:

(5) $$\begin{array}{ll} \;\text{Logit}(P(Y_{i}=1|Z_{i},X_{i}(k)))=\theta_{i}(k)=\gamma_{0}+\gamma Z_{i}+\beta X_{i}(k); & \; \end{array}$$

where *Z*_*i*_ and *X*_*i*_ are independent and were generated according to a standard normal distribution and the vector **X**_*i*_(*k*) was a transformation of a continuous variable *X*_*i*_. The sample size was set to be 100. We used 1000 replications for each simulation and 1000 samples for the resampling methods.

#### Dichotomous transformations

We only considered dichotomous transformations to explore a shape effect of the variable of interest. To obtain the best transformation, several cutpoints *c*_*k*_ may be tested. When epidemiological references are not available, a strategy based on the quantile of the continuous variable is most commonly applied. In this simulation we used the median for one dichotomous transformation. For two dichotomous transformations we used the first tercile as the first cutpoint, and the second tercile as the second cutpoint, and so on. This strategy is summarized in Table 1.

**Table 1:** **Strategy for dichotomous transformations: values of the cutpoints *c***_***k***_** according to the number of transformations (*q***_***α***_** represents the quantile of order *α *)**

**Number of transformations**	***c***_***1***_	***c***_***2***_	***c***_***3***_	***…***	***c***_***9***_
1	*q*_1/2_				
2	*q*_1/3_	*q*_2/3_			
3	*q*_1/4_	*q*_2/4_	*q*_3/4_		
⋮	⋮	⋮	⋮	⋮	⋮
9	*q*_1/10_	*q*_2/10_	*q*_2/10_	…	*q*_9/10_

Firstly, we investigated the Type-I error rate. For a replication, the rejection criterion of the null hypothesis (***β***_***k***_=0) was a *p*_*value*_ less than 0.05. Thus, for a simulation of 1 000 replications, the empirical Type-I error rate was the proportion of tests where the *p*_*value*_ was less than 0.05. Figure 1(a) shows the evolution of the Type-I error rate for dichotomous transformations. The *naive* method, without correction of the multiple testing, increases the Type-I error rate with the number of codings tried. For ten codings this error rate reached 0.27. The error rate calculated by the Bonferroni method decreased with the number of cutpoints. This correction was therefore too conservative whereas the exact method and resampling methods gave a Type-I error rate close to the nominal 0.05 value.

**Figure 1:**
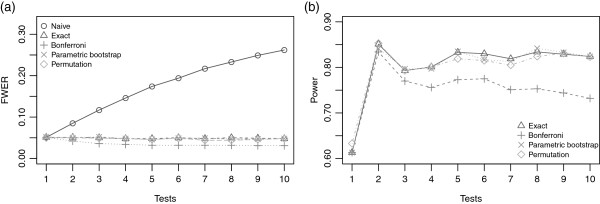
**(a) Type-I error rate for various numbers of cutpoints tried according to different methods; (b) power for *a threshold effect model * at the first tercile.** (*γ*_0_=−2.5,*γ*=1,*β*=2).

When information on the shape of the effect of the explanatory variable was unknown we investigated the power of the methods applied above. We studied the power for *a threshold effect model* with a cutpoint value at the first tercile. Figure 1(b) gives the power as a function of the number of cutpoints tried. The power of the exact and resampling methods are quite similar to one another, and higher than the Bonferroni method. The difference between these methods and Bonferroni method increases with the number of cutpoints. We also observed that the power was highest at two cutpoints (two transformations). This result, was in fact, expected since we used the first and second terciles respectively as cutpoints for each dichotomous transformation. Power increased again when trying five and eight codings due to the fact that one of these codings corresponded to the first tercile. To conclude, the simulation study with dichotomous transformations showed that the resampling methods provide similar results for the Type-I error rate control and the power as those seen with the exact method.

#### Categorical transformations with same number of classes

We considered here only categorical transformations with three classes. In this situation, the choices of the two cutpoints (noted $c_{k}^{1}$ and $c_{k}^{2}$) defining the categorical variables into three classes are also subjective. For this simulation study, our strategy was to attempt to find the most favorable transformation into three classes. This consisted of using the tercile of the variable for one transformation with two cutpoints (*c*11=*q*_1/3_ and *c*12=*q*_2/3_); for two transformations we add to the previous choice a transformation with the first quartile and the third quartile for the two cutpoints (*c*21=*q*_1/4_ and *c*22=*q*_3/4_). The global strategy until we obtain 10 transformations in three classes is presented in Table 2.

**Table 2:** **Strategy for the categorical transformations in three classes: values of the cutpoints (**$c_{k}^{1}$** and **$c_{k}^{2}$**) for all transformations**

**Number of transformations**	$c_{k}^{1}$	$c_{k}^{2}$
1	*q*_**1/3**_	*q*_**2/3**_
2	*q*_**1/4**_	*q*_**3/4**_
3	*q*_**1/4**_	*q*_**1/2**_
4	*q*_**1/2**_	*q*_**3/4**_
5	*q*_**2/5**_	*q*_**4/5**_
6	*q*_**1/5**_	*q*_**3/5**_
7	*q*_**3/5**_	*q*_**4/5**_
8	*q*_**1/5**_	*q*_**2/5**_
9	*q*_**1/5**_	*q*_**4/5**_
10	*q*_**2/5**_	*q*_**3/5**_

We investigated the Type-I error rate. Figure 2(a) shows the evolution of the Type-I error rate for categorical transformations in three classes. The results are similar to those we observed for dichotomous transformations. The Bonferroni correction was still too conservative, while resampling methods gave a Type-I error rate close to the nominal 0.05 value.

**Figure 2:**
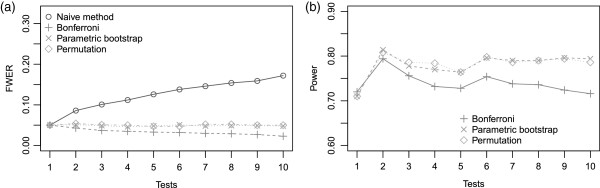
**(a) Type-I error rate for various numbers of categorical in three classes according to different methods, (b) and power for an effect of a categorical transformation in three classes defined by cutpoints at the first and third quartile.** (*γ*_**0**_=−1.25,*γ*_1_=1,*β*^*T*^=(2,1.8)).

Next we considered the power of the different methods when the simulated model was specified with a categorical transformation of the continuous variable in three classes defined by cutpoints at the first and third quartile. The two resampling methods gave similar results with a higher power than the Bonferroni method (see Figure 2(b)). The power was highest for two transformations. This result was also expected because, with the strategy presented in Table 2, the transformation into three classes with cutpoints at the first and third quartile is used.

#### Various categorical transformations

In this last simulation, we presented a more realistic situation where different kinds of transformations were used to investigate the effect of the variable of interest. We proposed trying different categorical transformations and varying the number of classes. The most natural method is to use a dichotomous transformation at the median for one transformation. For two transformations, we added the previous coding and a categorical transformation in three classes based on the tercile. For three transformations, we added the two previous codings and a categorical transformation in four classes based on the quartile, and so on. The strategy proposed in this simulation is presented in Table 3.

**Table 3:** Strategy for different categorical transformations: values of the cutpoints for all transformations

**Number of transformations**	$c_{k}^{1}$	$c_{k}^{2}$	***…***	$c_{k}^{9}$	$c_{k}^{10}$
1	*q*_**1/2**_				
2	*q*_**1/3**_	*q*_**2/3**_			
⋮	⋮	⋮	⋮	⋮	⋮
9	*q*_**1/10**_	*q*_**2/10**_	…	*q*_**9/10**_	
10	*q*_**1/11**_	*q*_**2/11**_	…	*q*_**9/11**_	*q*_**10/11**_

The results for the Type-I error rate were similar to the previous simulation case (not shown here). We then studied the power of the different methods when the simulated model is specified with a categorical transformation of the continuous variable in five classes defined by cutpoints at the quintile. We can see in Figure 3, that, in this situation, the parametric bootstrap method seems slightly more powerful than the permutation method. The resampling methods were also more powerful than the Bonferroni method. Finally, as expected, we can see that the power was highest for four transformations, where one of the transformations used corresponded to a categorical transformation with quintiles as cutpoints.

**Figure 3:**
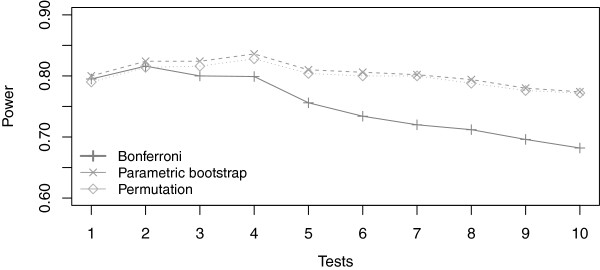
**Power for an effect of a categorical transformation in five classes defined by cutpoints at the quintile.** (*γ*_0_=−2.5,*γ*_1_=1,*β*^*T*^=(−1.3,−0.8,1.4,1.7)).

#### Robustness of resampling methods

We investigated the robustness of the resampling methods when the exchangeability assumption is violated. The data came from the model defined in (5) with two dependent variables *X*_*i*_(*k*) and *Z*_*i*_. The dependency between *X*_*i*_(*k*) and *Z*_*i*_ (formalized by the correlation ratio(*η*^2^)) was specified by the following model:

(6) $$Z_{i}=\beta^{*}X_{i}(k)+\varepsilon_{i};$$

where *X*_*i*_(*k*) is the binary coding of the *X*_*i*_ variable with a cutpoint at the median. The coefficient *β*^∗^ was computed according to *η*^2^ and the variance of *X*_*i*_(*k*) variable.We tested three different binary codings with cutpoints at the first, the second and the third tercile. The strategy is used for various values of the correlation ratio (*η*^2^) from 0 to 0.6.

The robustness of the permutation method when the exchangeability assumption is violated was evaluated with respect to the results of the exact method. For different correlation ratios (*η*^2^) we evaluated the control of the Type-I error, the power, the Mean Square Error (MSE) of the estimated *p*_*value*_ (*p*_*value*_ from the exact method was used as a reference), and the rate of good decision (same decision as for the exact method). These results are presented in Figure 4 and show the good behavior of the permutation method since the Type-I error is controlled at the level 0.05, the power is the same for all the methods, the rate of good decision is always greater than 0.97, and the MSE is very low. Moreover, the distributions of the estimated *p*_*value*_ are quite similar for different methods (not shown).

**Figure 4:**
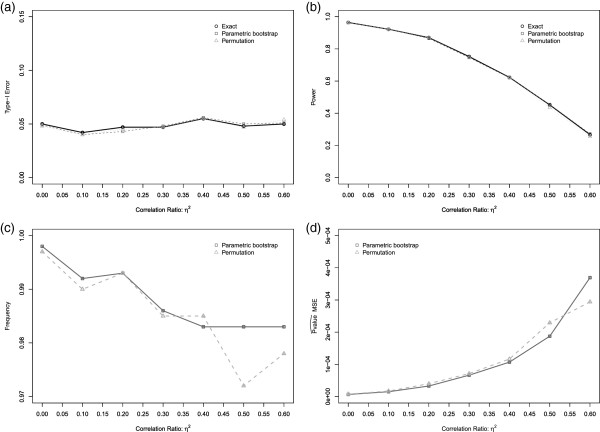
**Robustness of the *p***_***value***_** by Resampling methods for different values of correlation ratio *η***^***2***^** (*γ***_***0***_***=−2.5,γ***_***1***_***=1,β=2*): (a) Type-I error; (b) Power; (c) Correct decision rate; (d) Mean Square Error (MSE) of the **$\hat{p_{\text{value}}}$**.**

### Example: revisiting the PAQUID cohort example

In order to find the real association between the two variables of interest in the example described at the end of Background section, we applied our newly developed approach which combined different kinds of transformations. Liquet and Commenges [14] have proposed seven dichotomous and five Box-Cox transformations. However, their method did not allow for categorical transformations. We proposed to add, to the seven dichotomous and five Box-Cox transformations for this application, four codings in three classes and four codings in four classes. The best transformation appeared to be the dichotomous transformation of HDL-cholesterol with a cutpoint at the third quartile, as already found by Bonarek et al. [16]. The Bonferroni correction gave a *p*_*value*_ equal to 0.140, thus not significant for an *α* level at 0.05. The *p*_*value*_, which is given by both resampling based methods is 0.038. To conclude, it is important to chose a powerful method of correction, because in this context the *p*_*value*_ with no correction given by Bonarek et al. [16] was very optimistic (0.007), and the Bonferroni correction was very conservative, yielding an incorrect conclusion. The proposed approach based on the resampling procedure gave a result which was still significant and more realistic than the uncorrected *p*_*value*_.

## Discussion

In this paper, we have considered the problem of correction of significance level for a series of several codings of an explanatory variable in a Generalized Linear Model with several adjusting variables. The methods developed, based on resampling methods, enable us to consider categorical transformations as more flexible in order to explore the unknown shape of the effect between an explanatory and a dependent variable. The simulation studies presented above show, firstly, that the resampling method provides similar results for the Type-I error rate control and the power as those found with the exact method proposed by Liquet and Commenges [14] for dichotomous and Box-Cox transformations. Secondly, in the situation of categorical transformations, these simulations demonstrate the good performance of our proposed approaches. Finally we observed the robustness estimation of the *p*_*value*_ by the resampling methods. These methods can be easily generalized to other models, such as the proportional hazards model, and to potentially extend the work of Hashemi and Commenges [15] in the same context.

## Conclusion

To conclude, the methods developed, based on resampling, demonstrate good performances, and we have implemented different methods and different strategies of coding in an R package called CPMCGLM M (for Correction of the Pvalue after Multiple Coding in a Generalized Linear Model).

## Appendix

The package CPMCGLM has been developed in R, an open source statistical software available at http://www.r-project.org. The methods presented in this paper are available in the main function CPMCGLM() for Probit, Logit, Linear, and Poisson models. Briefly, the user can specify the transformations tested: Box-Cox, dichotomous or categorical transformations. Two options are possible for defining the cutpoints of the dichotomous and the categorical transformations: the user can either specify them, or the program will automatically use the strategy based on the quantile presented in the simulation study.

The main function provides the best codings according to the maximum test and minimum *p*_*value*_ procedures. For this coding, the different methods of correction of the Type-I error rate presented in this paper are provided. We present an illustration of the CPMCGLM function on a simulated dataset:

### 

## Competing interests

Both authors declare that they have no competing interests.

## Authors’ contributions

BL and JR developed the methodology, the R code, performed the simulation and the analysis on the dataset as well as wrote the manuscript. Both authors read and approved the final manuscript.

## Pre-publication history

The pre-publication history for this paper can be accessed here:

http://www.biomedcentral.com/1471-2288/13/75/prepub
